# Consensus Guidelines on Constipation in Adults in Pakistan

**DOI:** 10.12669/pjms.40.11.9687

**Published:** 2024-12

**Authors:** Amanullah Abbasi, Anton Vignaraj Emmanuel, Ghias Un Nabi Tayyab, Kashif Shafique, Lubna Kamani, Muhammad Bilal Nasir, Nazish Butt, Sajjad Hussain Sabir, Shahab Abid, Shahid Rasool, Tayyab Saeed Akhter, Zahid Azam

**Affiliations:** 1 Prof. Dr. Amanullah Abbasi Professor Emeritus Medicine, Head of Medical Unit, Civi Hospital, Karachi, Pakistan; 2 Anton Vignaraj Emmanuel MD University College Hospital, London, UK; 3 Prof. Dr. Ghias Un Nabi Tayyab, MBBS, FCPS, MRCP, FRCP, AGAF Dean, Faculty of Gastroenterology CPSP; 4 Prof. Dr. Kashif Shafique, MBBS, MPH, PhD Professor of Public Health, Dow University of Health Sciences, Karachi, Pakistan; 5 Prof. Dr. Lubna Kamani, MBBS, FCPS, MRCP, FRCP Professor of Gastroenterology, Liaquat National Hospital, Karachi, Pakistan; 6 Dr. Muhammad Bilal Nasir Assistant Professor Gastroenterology Al-Aleem Medical College, Lahore, Pakistan; 7 Dr. Nazish Butt, MBBS, FCPS. Associate Professor, Head of Gastroenterology Department, Jinnah Postgraduate Medical Center, Karachi, Pakistan; 8 Dr. Sajjad Hussain Sabir, MBBS, MD Gastroenterology, MACG (U.S.A.) Assistant Professor, Alkhidmat Hospital, Sahiwal, Pakistan; 9 Prof. Dr. Shahab Abid, MBBS, PhD, FCPS, FACG, FRCP Professor of Medicine – Gastroenterology, Aga Khan University, Karachi, Pakistan; 10 Prof. Dr. Shahid Rasool, MBBS, FCPS (Med), FCPS (Gastro), FRCP Professor of Gastroenterology and Hepatology Madina Teaching Hospital, Faisalabad, Pakistan; 11 Dr. Tayyab Saeed Akhter, MBBS, FCPS Consultant Medical Specialist and Gastroenterologist, Holy Family Hospital, Rawalpindi, Pakistan; 12 Prof. Dr. Zahid Azam, MBBS, FCPS (Gastro), FCPS (Med), FACG, M.Sc (Clinical Research) Professor of Medicine, Dow University of Health Sciences, Karachi, Pakistan

## INTRODUCTION

Constipation is a prevalent gastrointestinal disorder characterized by infrequent or difficult bowel movements, often associated with straining and the sensation of incomplete evacuation.^1^-^4^ Despite its high prevalence, constipation is commonly underreported, leading to delays in diagnosis and management. In Pakistan, as in many other regions, constipation remains a significant yet overlooked health issue, with implications for both individuals and the healthcare system. Addressing this requires a long-term approach focused on dietary, behavioral, and lifestyle modifications, guided by empathetic healthcare providers.

While functional constipation is generally defined as infrequent or difficult bowel movements without identifiable causes, the interpretation of “constipation” can vary widely.^5^ Patients often describe it in terms of symptoms like straining or pain during defecation, whereas healthcare providers may define it based on the frequency of bowel movements.^6^ This variation in perception highlights the importance of clear, context-specific guidelines.

Studies in Pakistan indicate a notable prevalence of constipation across different population groups. A study in Karachi reported that over half of hospitalized patients and their attendants, as well as a third of medical students, experienced constipation.^7^ Another study showed a similar prevalence among medical students, with a higher rate in females.^8^ Research in Lahore found that 14% of healthcare workers and medical students suffered from constipation, a figure comparable to global estimates.^9^ These findings are significant, as they highlight that constipation is not only a common issue globally but also a pressing concern in Pakistan. The higher prevalence observed among females may be due to factors such as hormonal fluctuations, diet, and lifestyle differences, which are also reflected in international studies.^10^-^12^ Understanding these local patterns is essential for clinical practice, as it underscores the need for gender-specific approaches in managing constipation. Furthermore, these findings call for the development of guidelines that address the unique socioeconomic and healthcare contexts of Pakistan, ensuring that interventions are both feasible and culturally relevant.

While international guidelines on constipation management provide a valuable framework, they often fail to account for the unique cultural, dietary, and socioeconomic factors that affect healthcare delivery in Pakistan. For instance, the high prevalence of constipation in Pakistani females may be influenced by cultural practices such as dietary restrictions, lower fiber intake, and reduced physical activity, which are less common in other regions. Additionally, access to healthcare and medications is often limited, especially in rural areas, making some of the treatment recommendations in international guidelines less feasible. These disparities highlight the need for context-specific guidelines that reflect the realities of the Pakistani healthcare system and the lifestyle of its population. By addressing these gaps, localized guidelines can provide more effective and practical solutions for managing constipation in Pakistan.

However, a lack of comprehensive community-based data for Pakistan underscores the need for targeted research and national guidelines. Given the high prevalence of constipation in Pakistan and the need for tailored guidelines, a structured methodology was adopted to develop consensus on its management. This evidence-based guidelines aims to provide healthcare providers with clear, practical recommendations to improve the diagnosis and management of constipation among the adult population in Pakistan.

## METHODS

### Development process:

The chronic constipation guidelines formulation was a multistep process and included selection of expert panel, four rounds of Delphi, analysis of qualitative data and the compilation of the final guideline recommendations.

### Expert Panel Selection:

The guideline development committee comprised of 11 experts from all across Pakistan, who were selected due to their extensive clinical experience and professional contributions relevant to the field. The expert panel encompassed a range of specialties including gastroenterology, internal medicine, public health, and clinical research.

### Method for Consensus Building:

The Delphi method, a structured, iterative process was selected for building consensus for guideline recommendations. The panelists conducted a comprehensive literature review on various aspects of constipation including its epidemiology, diagnosis, and management, and identified key areas of contention and emerging trends in the overall evidence on chronic constipation.

### Formulation of Preliminary Recommendations:

The panelists initially generated a list of recommendations deemed important for inclusion in the national chronic constipation guidelines for Pakistan. This preliminary set of recommendations based on scientific evidence were then collated into various themes. Panelists then rated the importance and feasibility of each recommendation and discussed the phrasing and scope of each recommendation. During Delphi rounds, comments and feedback provided by panelists were qualitatively analyzed to identify areas of divergence and consensus. Discrepancies and disagreements on various recommendation points were scrutinized to understand their underlying causes and guide further refinement based on existing scientific evidence and international best practices.

### Final Consensus and Agreement Threshold:

For the final consensus building, an agreement of >80% was considered appropriate to determine the consensus on various recommendation points.

## RESULTS

The recommendations that attained consensus were integrated into a structured and cohesive guideline document. The following recommendations were put forward:

### Types of Constipation:

Primary constipation, also called idiopathic constipation, has three types.^13^ It must be differentiated from Irritable-Bowel-Syndrome (IBS) as the symptoms might overlap but pain is an important differential symptom, and specifically in IBS the pain has a temporal association with the constipation.^3^ There are several causes of secondary constipation that may include dehydration due to reduced fluid intake, certain types of diseases that may cause metabolic derangements (Diabetes Mellitus, Hypothyroidism, hypercalcemia etc.). Some neurological or structural abnormalities, disorders, or myopathies may also be major reasons for constipation and should be managed accordingly.^8^

### Diagnosis of Constipation:

The key approach towards the diagnosis of constipation requires a comprehensive history, followed by a detailed physical examination including digital rectal examination (DRE). Literature from Pakistan suggests that there is a significant decline in the level of confidence & comfort in performing of DRE amongst the students and doctors.^14^ We recommend proper training of DRE amongst medical students and physicians (Table-I).

**Table-I:** Digital Rectal Examination in a Patients with Constipation.

Step	What to Do?	How to Do?	Why to Do?
1	Explain procedure to the patient	Inform about all the steps in detail	Relieve fear and anxiety of patient
2	Inspect the patient under good light in left lateral position with hips flexed	Gently retract the buttocks and inspect the anus	Check Symmetry, Scars, Skin abnormalities, Stool, Discharge of blood or pus, anal tags, warts, hemorrhoids, fistulous opening, abscess, condyloma
	Evaluate movements of anal muscles	Ask the patient to squeeze the anus	Look for Concentric movement of anus and perianal skin
	Inspect distal anus	Using a gauze piece at each side of anal orifice gently open the distal anus	Look for Fissure
	Inspect under strain	Ask the patient to strain	Look for Perineal descent syndrome**, Rectocele^***^, rectal or vaginal prolapse, Bulging hemorrhoids
3	Evaluate sphincter innervation	Check perianal skin sensations in all 4 quadrants using pinprick	Ask for the feelings
		Ano-cutaneous Reflex by stroking the perianal skin with cotton bud	Look for brisk contraction of perianal skin and the external anal sphincter.
4	Digital Palpation	Gently advance lubricated and gloved index finger	Feel mucosa and surrounding muscles/bones. Anal canal length, anorectal angle, Tenderness^$^, tone, mass, rectocele^***^, hard stool.
5	Maneuvers for Ano-rectal function	Ask patient to squeeze	Feel the increase in the anal sphincter tone
		With one finger in rectum, place other hand over abdomen and ask patient to push as if defecating	Evaluate push effort of abdominal wall, relaxation of anal canal and puborectalis muscles^#^, degree of perennial descent*, rectal mucosal prolapse
6	Finishing off	Take out the gloved finger	Look for blood/feces on the finger

* Normal perineal descent < 3.5 cm,

** Perineal descent syndrome > 4cm,

$ Excruciating pain suggests anal fissure, # tightening of muscles on push maneuver is indicative of paradoxical external anal sphincter contraction, suggesting anorectal dyssynergia.

*** Rectocele: A condition where the rectum bulges into the vaginal wall, often causing difficulty in bowel movements and incomplete evacuation.

**Fig.1:**
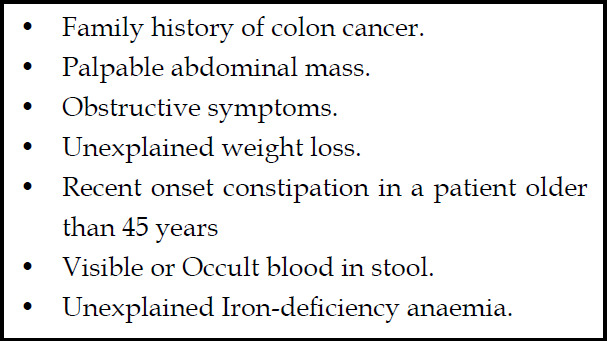
Red Flag Sign/Alarm Features.

In patients with recent onset symptoms, laboratory tests may point towards underlying possible etiology. This helps to differentiate between primary and secondary constipation and pick up any “Red Flag Sign” or “Alarm Features” to expedite the diagnostic pathway. (Fig.1). The guideline committee also recommends early colonoscopy in patients presenting with any of the alarm features.

### Rome IV criteria - Diagnostic criteria for Functional Constipation for Adults:

According to this criteria, functional constipation (FC) can be described as the occurrence of two or more of the following symptoms, encountered during more than one-fourth of defecation episodes:^4^,^15^

- Straining
- Lumpy or hard stools
- Sensation of incomplete evacuation
- Sensation of anorectal obstruction/blockage
- Manual manoeuvres to facilitate evacuation
- Fewer than three spontaneous bowel movements per week

For a diagnosis of constipation, these criteria must be present for the last three months, with symptoms beginning at least six months prior to diagnosis.^8^,^15^ While these criteria are useful in developing robust clinical trials, their applicability to the ‘real life’ clinical situation is limited by their complexity. In some patients, loose stools are occasionally present. The criteria helps identify individuals with constipation-predominant Irritable Bowel Syndrome (IBS-C) distinct from functional constipation since their treatment pathway is different (and outside the scope of this guideline).^4^,^8^ The guideline committee recommends considering associated abdominal pain, aggravated or relieved by defecation being the main presenting symptoms as the key guide to distinguish between IBS-C and FC. However, the criteria for the diagnosis of IBS neither excludes the existence of faecal evacuation disorder nor the slow transit constipation.^16^

In Pakistan, there can be a language barrier between physician’s and patient’s understanding of the symptoms, therefore apart from Rome criteria, the pictorial nature of the Bristol Stool Form Scale may be useful as it assists in self-evaluation of the patient’s perspective of constipation.^17^

**Table-II:** Types of protocols and their interpretation.

Type of Protocol	20 Radio-Opaque markers at time (in hrs)			Abdominal radio- graph at time (in hrs)		Interpretation
Western Protocol	0	24	48	72		≥ 20% of original markers
Ghoshal’s protocol	0	12	24	36	60	≥ 30 at 36 hrs and ≥ 14 at 60 hrs

All authors contributed eqally during compilation of guideline.

Secondary constipation may also be due to intra-intestinal or extra-intestinal causes, including mechanical impedance, pathologies of ano-rectum, myopathies and neuropathies, metabolic and endocrinological disorders, low-fiber food, a sedentary lifestyle, or psychological disorders.^8^,^16^ Certain medications like anti-depressants, anti-epileptics, anti-psychotics, calcium channel blockers, and anti-spasmodic drugs, could also be involved.^15^,^16^ Published data has shown that usually patients in Pakistan prefer to take laxatives and herbal supplements along with increased fluid intake and a high fiber diet.^17^

For a better understanding of constipation, the guideline committee recommends physiological assessment in patients who have already engaged in at least three months of lifestyle modification and first-line treatment with no improvement in their symptoms.

### Physiological assessment:

### i. Anorectal-Manometry and Balloon Expulsion Test:

- Anorectal manometry (ARM) studies assess:
- Sphincter tone at resting and during squeeze,
- Recto-anal inhibitory reflex,
- Rectal sensation,
- Change in anal and rectal pressure during an attempt to defecate, and
- The ability to the patient to expel a 50 ml water-filled balloon.

ARM is difficult to access in Pakistan and is currently available in only limited centers. However, the Balloon Expulsion test can easily be performed as a separate test as well as on the bedside in the left lateral or sitting position, where a balloon tied with a thin catheter is placed in the rectum. By convention, the balloon is filled with 50 ml water and the patient is directed to expel it, noting the time taken to do so. The London Protocol Version 1.0 is an attempt to standardize the manometric parameters but requires more studies and validation of protocol.^18^ For patients with chronic constipation who fail to respond to first-line therapy, ARM can be considered, however, ARM can over-diagnose anorectal Dyssynergia.^19^ The guideline committee encourages local studies for a better understanding of the validity of ARM in the Pakistani population.

### ii. Defecography:

Defecografy can be performed by using barium or magnetic resonance imaging. Patients with normal ARM and Balloon expulsion may undergo defecography testing. The yield is expected to be higher in structural rectal abnormalities like Rectal prolapse, recto-anal intussusception, enterocele and excessive perineal descent.^20^ A Study from India have demonstrated that multiple tests including ARM, Balloon expulsion and defecography have better yield than single tests performed for these patients.^21^ However, the practicality, availability and cost implications need to be factored into any discussion in the local setting.

### iii. Colonic Transit Studies:

Colonic transit studies can be reserved for patients with normal anorectal testing or those with persisting symptoms despite treating anorectal disorder. Colonic transit studies can be performed either with the help of radio-opaque markers, scintigraphy or wireless motility capsule to diagnose the Slow Transit Constipation (STC). The radio-opaque marker test is the simplest and cheapest option and hence preferred. However, colonic transit studies can be delayed even in evacuation disorders, the test lacks standardization, and since local data is unavailable, hence we are dependent on Western data for the normative values. An Indian study has shown that the Western protocol for colonic transit doesn’t hold good evidence for their population as they have a faster transit and they have modified their protocol.^22^

The quality-of-life aspects faced by the patients particularly in the elderly group and their burden on the society should also be considered. Findings from a published Pakistani study suggested that the majority of patients had normal transit, and medications that reduce transit time might not be useful in managing such individuals. Hence, to optimize treatment program, we need to better understand patient’s perspective.^17^

### Treatment of Constipation:

The management of primary constipation primarily depends on the type, and involves a combination of lifestyle modifications, dietary adjustments, and, if necessary, medical interventions, biofeedback, and retraining.^23^,^24^ Treatment should be cost-effective with emphasis on improving patients’ overall quality of life.

### i. Lifestyle modifications^*^:

- Regular physical activity to improve bowel movements and health of the digestive tract. Aim for at least 150 minutes of moderate exercise per week, as suggested by health organizations.
- Do not ignore the urge to defecate.
- Chronic stress can affect digestive function. Techniques including deep breathing exercises, meditation, or yoga may be effective in reducing stress. Similarly, engagement in mindfulness can help reduce anxiety and overall quality of GI health and well-being.
- Ensure 7-9 hours of quality sleep every night, since sleep disruption can affect bowel regularity.

* Consider other factors (regular routine, toilet position, mobility and exertion, etc.).

### Dietary modifications:

- Eat a diet rich in fiber obtained from whole grains (Oats, brown rice, and whole-grain bread), fruits (Apples, banana, oranges and pears (with skin when possible), vegetables (Leafy greens, carrots, broccoli, and Sprouts), legumes (Beans, lentils, chickpeas), and nuts (Almonds, chia seeds). Consume at least 25-30 grams (≈8-10 teaspoons) of fiber daily.
- Ensure plenty of daily water-intake to prevent dehydration and assist in softening the stools. Aim for atleast 8 cups/glasses (64 ounces) per day.
- Reduce consumption of highly processed foods, which are often low in fiber and nutrients.
- Limit fat-rich food.

### ii. Medications:

Patients should be directed to use medications under the guidance of a healthcare professional, especially if the constipation is chronic or severe. Following laxative medications can be used to treat constipation:

- ***Bulk-forming Agents (Fiber Supplements):*** The mechanism of action of these medications entails increasing the bulk and water content of the stool, to make it softer and easier to expel. Examples include psyllium (Metamucil) and methylcellulose (Citrucel).
- ***Stool Softeners:*** An example of stool softener is Docusate sodium (Colace), which helps to soften the stool by increasing the water content. They make it easier for stool to move through the intestines.
- ***Osmotic Laxatives:*** These agents work by directing more water into the intestines that assists in softening the stool, and promotes bowel movements. Examples include polyethylene glycol (MiraLAX), lactulose, and magnesium hydroxide (Milk of Magnesia).
- ***Stimulant Laxatives:*** Stimulant laxatives help stimulate the muscles of the intestines, promoting bowel movements. Examples include Sodium Picosulphate (Laxoberon) bisacodyl (Dulcolax), and senna (Senokot).
- ***Lubricants:*** Lubricant laxatives, like mineral oil, coat the stool and the intestinal lining, making it easier for stool to pass.
- ***Enemas and Suppositories:*** These are used for more immediate relief. Enemas involve introducing liquid into the rectum to stimulate bowel movements, while suppositories are inserted into the rectum to soften and stimulate stool evacuation.
- ***Prescription Medications:*** In cases of severe or chronic constipation that have not responded to other treatments, a healthcare provider might prescribe medications like linaclotide (Linzess) or lubiprostone (Amitiza), which work by increasing fluid secretion and motility in the intestines.
- ***Prokinetic Agents:*** These medications improve the movement of the intestines, which can help relieve constipation. An example is prucalopride.
- ***Medications for Specific Conditions:*** In case the constipation is a consequence of an underlying medical problem, such as IBS or opioid-induced constipation, the healthcare provider might recommend specific medications tailored to the condition. For instance, Picosulphate is the drug of choice during pregnancy.^25^
- In the clinical decision-making process for patients presenting with constipation, physicians should incorporate economic cost of management and parameters related to quality of life etc. This holistic approach is supported by the broader understanding that healthcare decisions extend beyond the purely medical domain.^26^ Evidence does support certain drugs better improve the quality of life in individuals with chronic functional constipation.^27^,^28^
- ***Biofeedback for constipation:*** The neuromuscular training by biofeedback technique aims to achieve regularity in the normal pattern of defecation. The biofeedback is learning process in which we use different tools that are based on operant conditioning techniques.^29^ In dyssynergic defecation, there are two goals of neuromuscular training:
- To rectify the incoordination between the abdominal, rectal, pubo-rectalis and anal sphincter muscles to ensure a normal and complete evacuation of the bowel
- ***Correction of Dyssynergia:*** Dyssynergia refers to a condition where the muscles involved in defecation, particularly the pelvic floor and abdominal muscles, do not coordinate properly, leading to difficulties in bowel movements. Correction of dyssynergia involves training to improve the abdominal push effort, which engages the diaphragmatic muscle, while simultaneously relaxing the pelvic floor muscles. This is typically achieved through manometric-guided techniques, a method that uses pressure measurements to guide therapy. Following this, patients undergo simulated defecation training to enhance coordination during bowel movements, ultimately improving the efficacy of the defecation process. The following phases delineate the proposed protocol for biofeedback training:

***Phase I:*** Evaluation and enrolment

Interview, stool diary

Colonic and ano-rectal function test

Symptoms assessment

Breathing exercises involving diaphragm

Use of laxatives, timed=toilet training.

***Phase II:*** Active phase of biofeedback therapy.

Visual/Auditory/Verbal Feedback techniques-biweekly for six sessions (with each session lasting 60 min).

Use of home devices

***Phase III:*** Reinforcement of training at six weeks, three, six and 12 months

Rectoanal Coordination

Simulated Defecation training

Enhancing the sensory perception in rectum of individuals with impaired rectal sensation by intermittent inflation of balloon in rectum.

### iii. Surgical Treatment:

Cases of refractory constipation, with delayed intestinal transit and not responding to any other treatment, may resort to surgical treatment.^1^ An individual-centred approach must be followed to ensure that each individual is being managed by experienced and well-trained surgeons and in those institutes that are equipped with all the required tools and facilities to ensure proper and complete recovery.

### When to refer:

Several factors may result in referral of a patient with constipation from a primary care practitioner to a gastroenterologist. These factors may include:

- Inadequate response to the empiric treatment of constipation
- Worsening of symptoms of constipation despite continuous treatment
- Unintended loss of weight (more than 10% in three months)
- Persistent unexplained change in bowel habits
- Palpable mass in lower right abdomen or the pelvis
- Blood in stools
- A family history of inflammatory bowel disease or malignancy of colon
- Rectal tenesmus
- Iron deficiency anemia
- Screening colonoscopy over 45 years of age
- New-onset symptoms after 45 years of age
- Positive fecal occult blood test
- Cachexia

## CONCLUSION

These guidelines provide a comprehensive framework for health professionals for the diagnosis and management of constipation in Pakistan. It emphasizes the importance of a thorough history, physical examination, and targeted investigations to differentiate between primary and secondary constipation. The guidelines also highlight the role of lifestyle modifications, dietary adjustments, and pharmacological interventions in the treatment of constipation. The key take home messages include:

- A structured approach to the diagnosis of constipation, incorporating Rome IV criteria and the Bristol Stool Form Scale.
- Early colonoscopy in patients with alarm features.
- Physiological assessment in patients with persistent symptoms despite first-line treatment.
- PIndividualized treatment plans based on the underlying cause and patient socio-economic factors.
- Referral to a gastroenterologist in cases of refractory constipation or concerning symptoms.
- By adhering to these guidelines, healthcare providers can improve the diagnosis, management, and quality of life for patients with constipation in Pakistan.
